# Use of Deep Learning Networks and Statistical Modeling to Predict Changes in Mechanical Parameters of Contaminated Bone Cements

**DOI:** 10.3390/ma13235419

**Published:** 2020-11-28

**Authors:** Anna Machrowska, Jakub Szabelski, Robert Karpiński, Przemysław Krakowski, Józef Jonak, Kamil Jonak

**Affiliations:** 1 Department of Machine Design and Mechatronics, Faculty of Mechanical Engineering, Lublin University of Technology, Nadbystrzycka 36, 20-618 Lublin, Poland; a.machrowska@pollub.pl (A.M.); j.jonak@pollub.pl (J.J.); 2 Section of Biomedical Engineering, Department of Computerization and Production Robotization, Faculty of Mechanical Engineering, Lublin University of Technology, Nadbystrzycka 36, 20-618 Lublin, Poland; 3 Chair and Department of Traumatology and Emergency Medicine, Medical University of Lublin, Staszica 11, 20-081 Lublin, Poland; przemyslaw.krakowski84@gmail.com; 4 Department of Clinical Neuropsychiatry, Medical University of Lublin, 20-439 Lublin, Poland; k.jonak@pollub.pl; 5 Department of Computer Science, Lublin University of Technology, 20-618 Lublin, Poland; 6 Faculty of Mechanical Engineering, University of Zilina, Univerzitna 1, 010 26 Zilina, Slovakia

**Keywords:** deep learning networks (DLN), statistical modeling, prediction, mechanical parameters, compressive strength, bone cement

## Abstract

The purpose of the study was to test the usefulness of deep learning artificial neural networks and statistical modeling in predicting the strength of bone cements with defects. The defects are related to the introduction of admixtures, such as blood or saline, as contaminants into the cement at the preparation stage. Due to the wide range of applications of deep learning, among others in speech recognition, bioinformation processing, and medication design, the extent was checked to which it is possible to obtain information related to the prediction of the compressive strength of bone cements. Development and improvement of deep learning network (DLN) algorithms and statistical modeling in the analysis of changes in the mechanical parameters of the tested materials will enable determining an acceptable margin of error during surgery or cement preparation in relation to the expected strength of the material used to fill bone cavities. The use of the abovementioned computer methods may, therefore, play a significant role in the initial qualitative assessment of the effects of procedures and, thus, mitigation of errors resulting in failure to maintain the required mechanical parameters and patient dissatisfaction.

## 1. Introduction

The biomaterials used in medicine that are referred to as cements are used as binding materials in orthopedic surgery and dentistry. The origins of these materials date back to the early 1960s when Charnley performed hip alloplasty using polymethylmethacrylate (PMMA) cement to fix a femur [1]. Such bone cement continues to this day to be produced from a mixture of a monomer (methyl methacrylate, MMA) and a polymer in the form of pre-polymerized polymethyl methacrylate. The process takes place in the presence of an initiator, an activator, and a stabilizer [2,3]. In orthopedic surgery, these materials are most often used in hip or knee alloplasty and when it is necessary to fill a cavity due to missing bone tissue in complicated fractures or in cases of neoplastic lesions in order to strengthen the internal structure [4].

Bone cement, however, is most commonly used in arthroplasty of large lower limb joints, such as the knee or the hip. The number of procedures is increasing annually, with more than 1 million hip replacement surgeries performed each year worldwide [5]. According to the Swedish Annual Report on Prosthesis Implantations (SKAR) for 2019, over the last four years, the number of primary knee replacement surgeries increased by 17%, and 92% of knee prostheses are fixed with bone cement [6]. In the majority of publications, the opinions concerning the knee joint endoprosthesis procedure are very good among both doctors and patients, as they result in long-term subsidence or a significant reduction of pain or in the restoration of the range of motion [7,8,9]. However, the 15-year survival of knee joint prostheses fixed with bone cement is estimated at 90% [8]. In the United States alone, the number of revision arthroplasty procedures is expected to increase by 600% by 2030 [10]. This means that a significant proportion of patients will have to undergo a revision arthroplasty procedure, which is a worse prognosis than the original joint arthroplasty procedure [11]. Many factors, such as prosthesis placement, infections, and the mechanical strength of bone cement, which is the only element that connects the bone with the prosthesis, have an impact on the number of revision arthroplasty procedures. The environment surrounding the cement after the implantation is aggressive, which has a significant impact on the speed of aging of bone cement and, consequently, on the speed of changes in its mechanical properties [4,12].

The long-term properties of bone cement, including fatigue behavior, the viscoelasticity of creep, and stress relaxation, are central to the success of cemented hip replacement [13]. The bone/cement interface is the key to the survival of a total hip replacement (THR) [14,15,16]. The mechanical properties of bone cement are influenced by a number of factors, one of them being the method of preparation (mixing) [17]. Impurities introduced during its implantation, such as blood or bone tissue residues, as well as deviations from the recommended ratio of components also greatly affect its mechanical parameters [12,18]. The contamination of cement with blood causes a decrease in its strength properties and a discontinuity of its structure [12,19,20]. The basic strength properties, resistance to cracking, fatigue strength, and creep resistance of bone cement depend on its preparation process and its implantation, as well as on the influence of the environment of the human body [21,22,23].

With a view to obtaining material of improved mechanical and biological properties, the effect of introducing modifying additives, including biodegradable additives, has been widely investigated (cellulose, chitosan, magnesium, polydioxanone, or tricalcium-phosphate) [24], antibiotics (Ciprofloxacin and Vancomycin) [25], hardening solutions (hardystonite (HT; Ca_2_ZnSi_2_O_7_) mixed with brushite (B; CaHPO_4_·2H_2_O), calcium phosphate, and citrate liquid) [26], and antimicrobial particles of silver sodium hydrogen zirconium phosphate (S–P) [27].

An analysis of changes in mechanical parameters is extremely important from the viewpoint of implantation and estimation of implant lifetime and in the design of modern materials. However, it is often impossible to carry out a large number of physical tests due to high costs. A possible solution in this case is the use of predictive models, which enable the determination of the dependence between particular parameters and properties using earlier experimental data [28]. However, there are better alternatives, such as the finite element method (FEM) [29,30,31] or the boundary element method (BEM) and an analytical approach. The models developed in this way may indicate the most promising direction for research and help reduce the number of physical tests, thus significantly bring down the costs of the research [28,32,33,34].

Keeping in mind the abovementioned facts, first of all, the impact of changes in the strength of bone cements when various admixtures, both intended and accidental contaminants, is known. One must not forget the significant impact of the material properties of the cement on the failure of the reconstruction, and above all—on all the inconveniences resulting from revision procedures (re-risk and costs of surgery, inconvenience to the patient, etc.)—that the analysis of the influence of the extent of contamination with pollutants present in the operating field on the selected strength characteristics of the cement seems to be the most justified. Considering the fact that bone cement is rather expensive, surgical revision procedures carry additional risk to the patient, and any destructive experimental studies are long and cost-intensive, an attempt was undertaken to employ the available tools (deep learning networks and statistical modeling) to predict strength changes, as well as assess the effectiveness of the tools in question.

## 2. Materials and Methods

The experimental research included compression strength tests on bone cement samples to determine their mechanical properties depending on the degree of contamination. Based on the results of the experimental research, statistical models were developed and changes in the compressive strength of bone cements were predicted as the contamination increased. The obtained model results were compared with the results of the experimental research.

### 2.1. Material and Sample Preparation

Samples made of the DePuy CMW 3 Medium Viscosity Bone Cement were tested for compressive strength. The samples that underwent the compression test were prepared in accordance with the guidelines of the International Standard ISO 5388:2002 [35]. The samples were cylindrical, their height was 12 ± 0.1 mm, and their diameter was 6 ± 0.1 mm. In order to determine the effect of contamination of the cement structure with 0.9% saline solution and with blood, at the stage of components’ joining, 1%, 2%, 4%, 6%, 8%, and 10% of the contaminants were introduced into the samples. The samples prepared in this way were seasoned in Ringer’s solution at 23 °C and tested before the seasoning, 24 h after they were made, and after 10, 20, and 30 days in the solution. The study was approved by the Scientific Research Ethics Committee of the Lublin University of Technology (No. 5/2016 of 19 December 2016). Detailed information on sample preparation is provided in publications [12,18].

### 2.2. Mechanical Testing 

Compression strength tests were performed using the MTS Bionix-Servohydraulic Test System (Eden Prairie, MN, USA), which comprised a strength-testing machine with a dedicated MTS TestWorks software (Eden Prairie, MN, USA). The compression speed was selected in accordance with ISO 5388:2002 and was equal to 20 mm/min. Each of the sample series tested consisted of a minimum of five samples, according to the abovementioned standard. Detailed information on the compression strength test is provided in publications [12,18].

### 2.3. Deep Learning Networks’ Algorithms

The problem of the prediction of mechanical properties depending on the content of admixtures continues to be an interesting research area [36,37,38]. However, it should be noted that the authors of the abovementioned publications had a slightly different approach to the problem of prediction. The prediction was performed on a single set of data, from which strength relationships were predicted. The present paper provides for one or, in the second variant, two full sets of data in the form of full-strength characteristics. Such formulation of the problem determined the choice of the neural network algorithm: DLN LSTM (Deep Learning Networks Long Short-Term Memory).

The practical experience [39,40] gained over time showed that the capabilities of classical recursive neural networks are of limited use due to the latency between important elements of the time series. Algorithms that store and transmit full information about the analyzed time series may be inefficient due to the need to adapt to a specific application and the difficulty in the scalability of the model for long-term time relationships [41].

The Long Short-Term Memory (LSTM) is an architecture of recursive neural networks that is widely used in the field referred to as deep learning. The main assumption of the LSTM is to use a memory unit (cell) that stores its state over time, with the simultaneous presence of special nonlinear controllers in the form of gates, which are responsible for the correct flow of information. Gates are responsible for the proper flow of information between memory cells. Originally [42], three gate structures were introduced: an input gate, an output gate, and a forget gate that resets the state of the memory. Later [43], the LSTM architecture was simplified and reduced to the Gated Recurrent Unit (GRU), where the number of gates was reduced and the tasks of the forget gate were transferred to the output gate.

In order to determine the characteristics of the stress–strain relationship, for samples with modified input parameters in the form of defects in the composition of the mixture, a neural network algorithm referred to as deep learning, which contained a large number of hidden layers and gate structures, was used. The LSTM architecture contained in the MATLAB Deep Learning package R2020b with the Adam solver was used [44,45].

The calculation phase included predicting time series using the LSTM network. Stress values as a function of the deformation, obtained for individual samples, were declared as the input data. The pretraining data was preprocessed: The raw data set was reduced by removing the results of negligible low stress (the start of the linear range) together with the artifacts that resulted from the travel and the initial contact of the strength-testing machine with the samples. The length of the time series was then resampled due to the need to keep the same data lengths for each of the disturbance variants under further analysis. The data sets for each of the variants of the examined cases (disturbances) were combined into sequences: for blood admixture and saline content at the levels 0, 1, 2, 4, 6, 8 and 10% of the admixture.

Based on the data of the compression strength of cement with introduced contaminants in the share of 0, 1, 2, 4, 6, and 8%, constituting the training data set (72% of all data), separately for the addition of blood and saline, the full compression strength characteristics were predicted for the 10%-contaminated bone cement. In the second variant, two full compression strength characteristics were predicted from the training set obtained from cement characteristics obtained at the contamination of 0, 1, 2, 4, and 6% of the total cement volume. The obtained characteristics constituted the basis for the forecast of contamination in the shares of 8 and 10% by volume, respectively.

The predicting procedure using deep learning networks was carried out in two variants. In the case of the averaged input data, the data from all the experimental trials were averaged, separately for each contamination level and contaminant type. The second variant was the study based on random combinations of input time series: Adjusted raw data from each of the conducted experimental trials were used separately.

The data were standardized to prevent discrepancies during the learning process. The LSTM network was built to predict the value of each of the subsequent time steps. Data vectors without the last step were declared as the predicators. As a part of the declaration of the network architecture, the number of hidden units was declared to be 50 and the number of iterations covered 150 epochs. So as to prevent degeneration of the model describing the examined phenomena, a gradient threshold was declared and a dropout option was used to prevent the model from being attached to the analyzed data.

The next stage of the research was to obtain the force value at the point specified in ISO 5833: 2002 [35]: A destructive force, the load shifted by 2% or load at the upper yield point (whichever occurred earlier), and transform it into the compressive tension. In the final stage, the forecast results were compared against the real experimental data. As part of the assessment of the quality of the obtained forecasts, the values obtained experimentally were compared with the data obtained using the deep learning networks tool.

### 2.4. Statistical Modeling

In order to predict changes in selected strength parameters, statistical analyses were carried out. These led to the generation of mathematical models that could describe changes in compression strength and microhardness of bone cement material enriched with physiological salt or human blood injected at a certain percentage concentration by weight. Developing such a model from experimental research results will enable estimating the value of these parameters for other admixture cases, e.g., for larger quantities of contaminants. To that end, an attempt was made to determine such models by analyzing and fitting typical variations (linear, polynomial function, different degrees). The accuracy of fitting of the selected models to the empirical results and the accuracy of prediction of the model were analyzed by comparing the predicted results with the actual ones in two variants: a model prepared from input data defined in the admixture range of 0–8% and 0–10%. The modeling was conducted using Statistica software (data analysis software system), version 13, from TIBCO Software Inc. (2017) (Palo Alto, CA, USA), and Microsoft Excel 2019 (Redmond, WA, USA).

## 3. Results

### 3.1. Deep Learning Neural Networks

The analyses enabled obtaining results for two cases: a prediction variant for the last series and two last series of tests. The time series specific for disturbances in the form of changed proportions of bone cement components and blood and saline admixtures were studied. The results are presented in Section 3.1, for the tests of the last variant of the disturbance, and Section 3.2, for the tests of the last two variants. The results of the analyses were compared with the actual results obtained during the conducted experimental research to estimate the effectiveness of the deep learning algorithms in the construction of models that describe the relationship between the proportions of the components and the strength properties of bone cements.

#### 3.1.1. Modeling Single Characteristics

**Figure 1:**
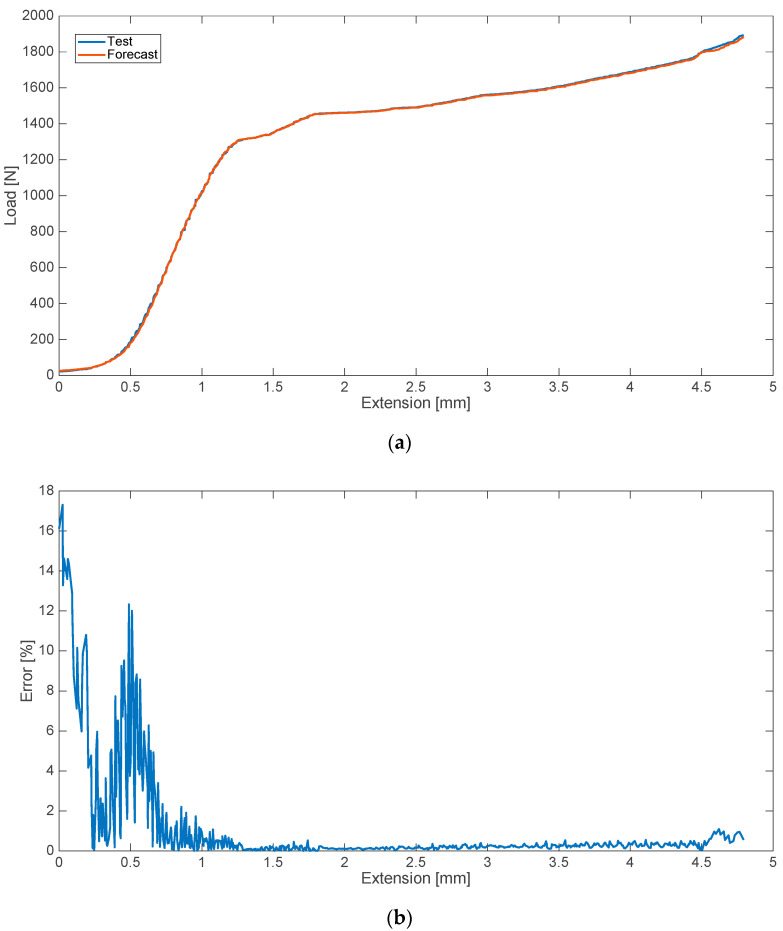
Strength characteristics (**a**) and error values (**b**) for 10% blood admixture.

The results were obtained for three variants of bone cements’ composition disturbances. The first of the analyzed cases was a disturbance consisting of the addition of 10% of blood. The results of the algorithm in comparison with the actual data obtained and the relative error values, as a function of deformation, are shown in Figure 1.

**Figure 2:**
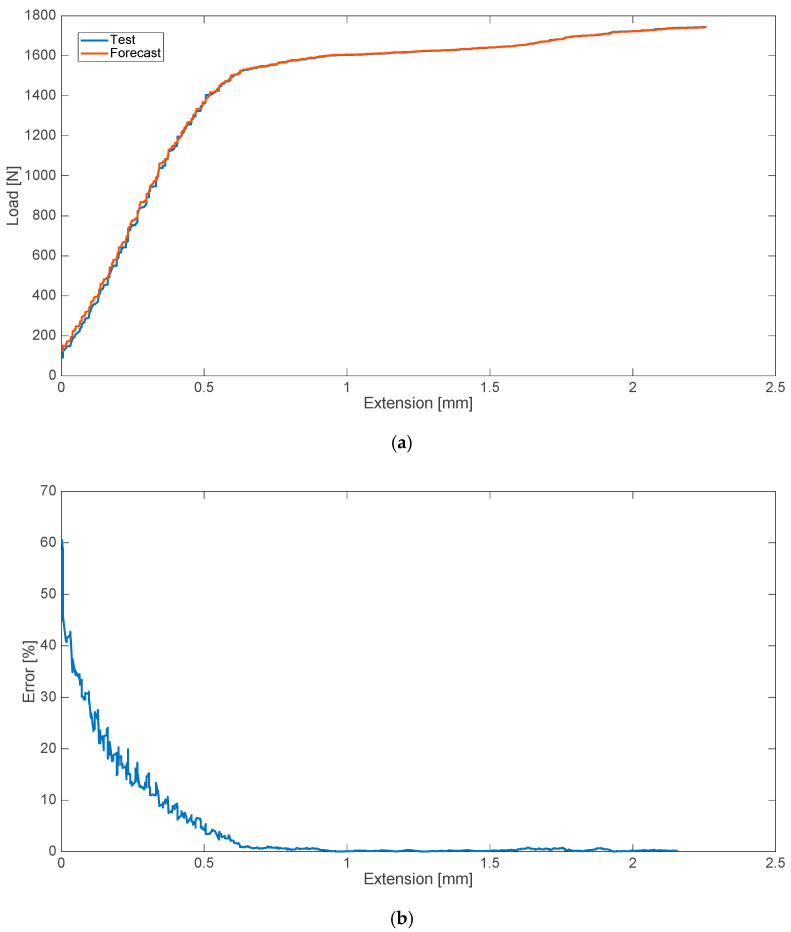
Strength characteristics (**a**) and error values (**b**) for 10% saline admixture.

In addition, the cases of disturbances in the form of admixture of physiological salt were analyzed. As in the case of blood admixtures, analyses were performed to predict the strength characteristics of the material with the 10% admixture of saline. The results of the analyses and tests with relative errors are shown in Figure 2.

#### 3.1.2. Modeling of Double Characteristics

**Figure 3:**
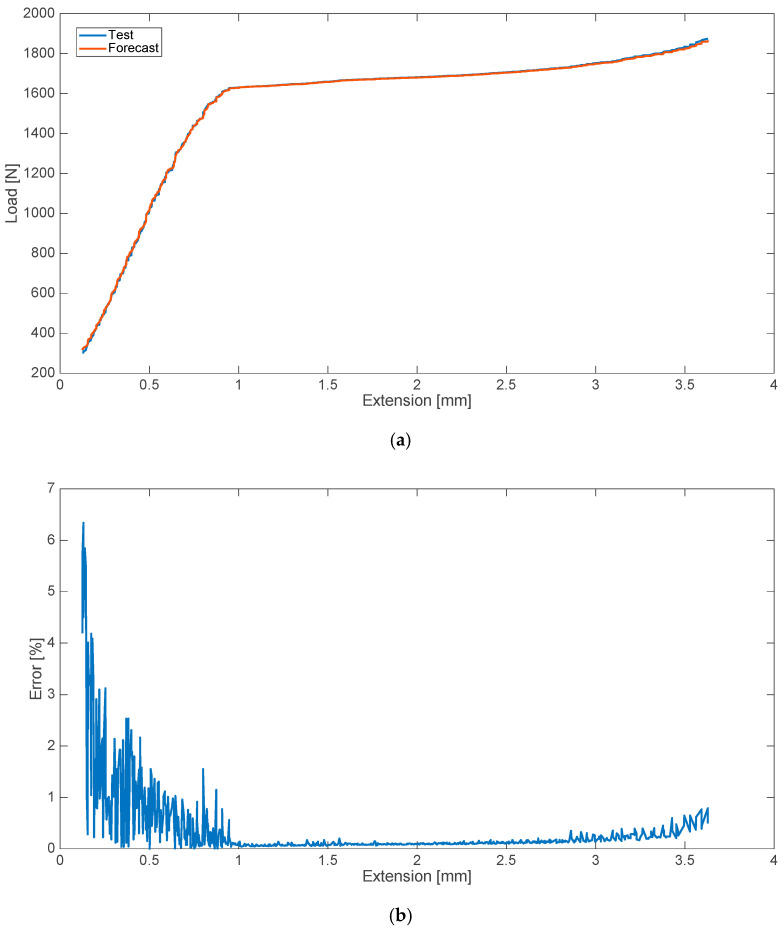
Strength characteristics (**a**) and error values (**b**) for 8% blood admixture.

**Figure 4:**
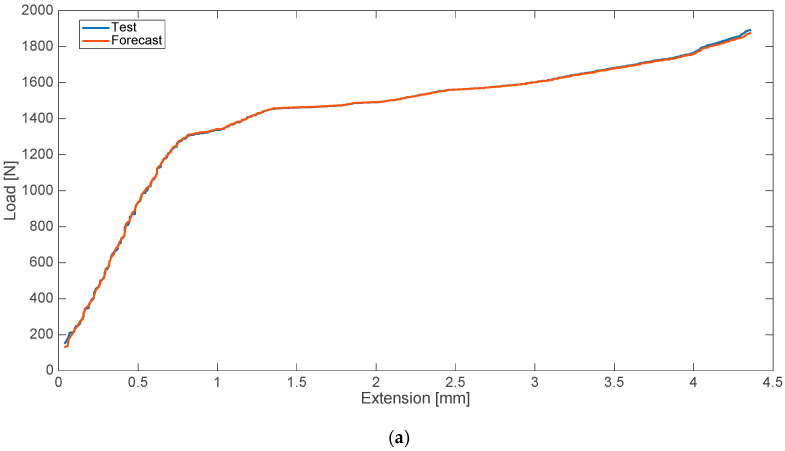

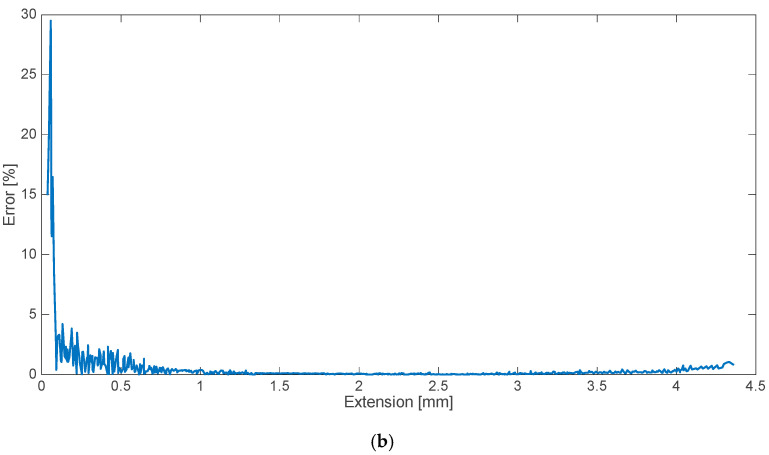
Strength characteristics (**a**) and error values (**b**) for 10% blood admixture.

The research conducted in the second variant included the determination of two strength characteristics (two data series). In the case of a blood admixture, these were the characteristics corresponding, in turn, to the disturbances in the form of the 8% (by weight) contamination of cement with human blood (Figure 3) and an admixture of 10% (Figure 4). The relative error values for the analyzed cases are presented on the side.

**Figure 5:**
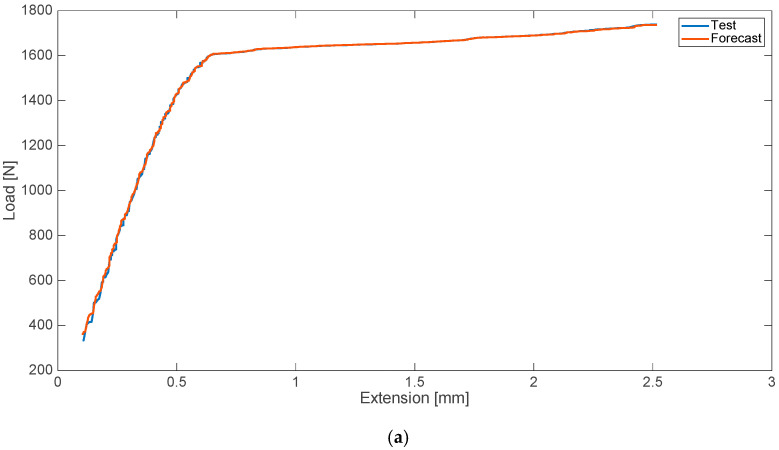

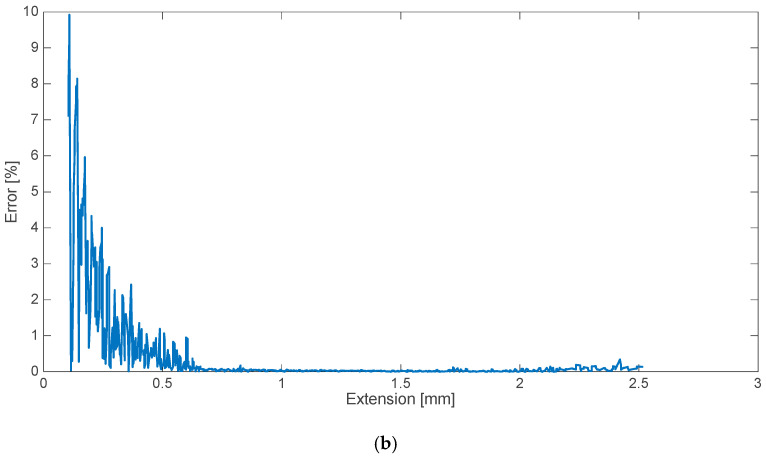
Strength characteristics (**a**) and error values (**b**) for 8% saline admixture.

**Figure 6:**
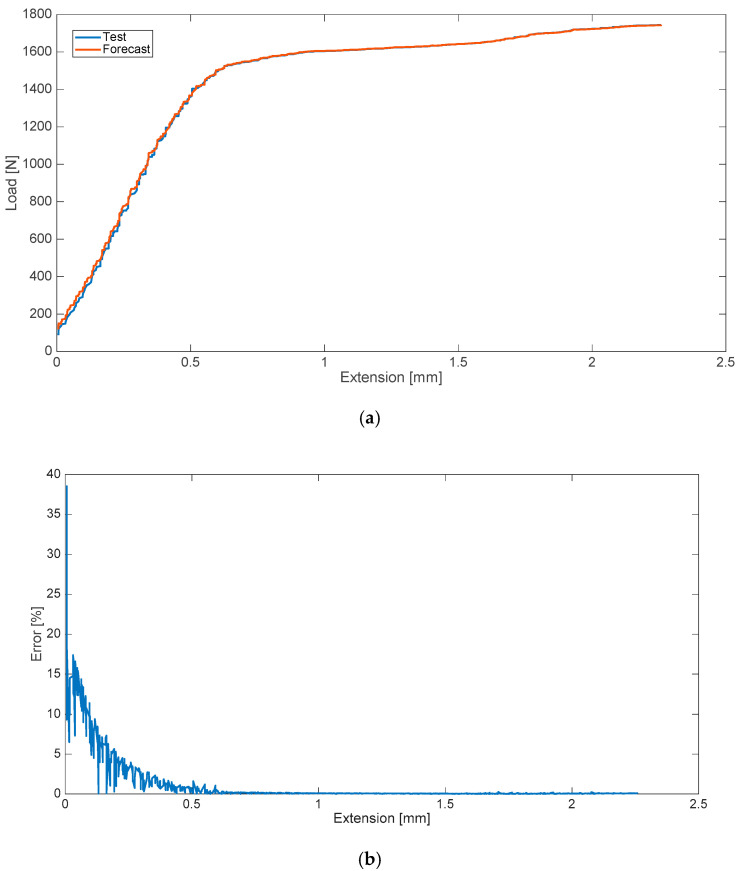
Strength characteristics (**a**) and error values (**b**) for 10% saline admixture.

The same procedure was applied to the saline admixture case. An estimation was made to determine the characteristics corresponding to the admixture ratio of 8% (Figure 5) and 10% (Figure 6) and the values of the prediction error were presented.

### 3.2. Mathematical Modeling of Strength Characteristics of Cements

Based on the results of the experimental destructive compressive strength testing of cement samples, uncontaminated and contaminated with blood or saline on the level of 1%, 2%, 4%, 6% and 8%, mathematical models (linear, polynomial, higher polynomial, and additionally exponential) were developed. Also, an attempt was made to prepare a model based on the 0–6% admixture teaching set. The model was based on the results of experimental research for uncontaminated and contaminated samples for the 0%, 1%, 2%, 4% and 6% levels.

#### 3.2.1. The 0–8% Model

**Figure 7:**
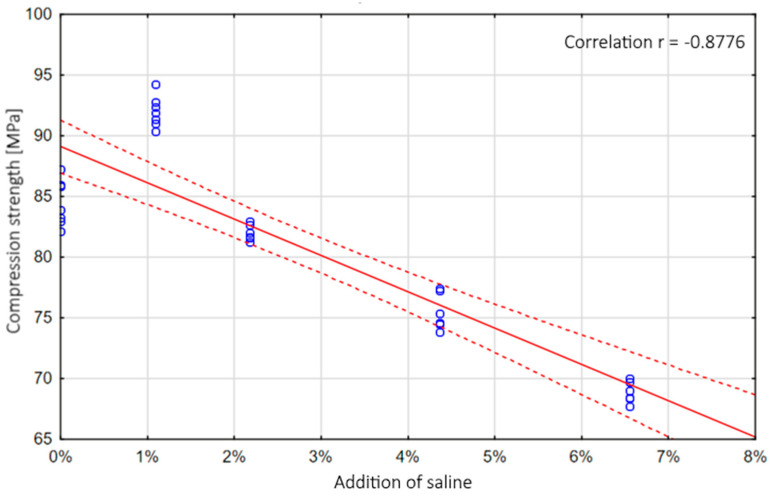
Comparison of the correlation between the admixture level and the compression strength of samples contaminated with saline up to approx. 8% by weight.

The model was based on the results of experimental research for uncontaminated and contaminated samples for the 0–8% level. Figure 7 shows an example diagram of the correlation between the degree of contamination and the compression strength. Table 1 shows the obtained parameters of the polynomial models, along with the coefficient of determination R^2^, which describes the degree to which the models fit the actual data, while Table 2 shows the parameters of the exponential models.

On the basis of the generated models, an attempt was made to predict the values of compression strength of cement with 10% admixture of both the contaminants (separately). Then, the obtained values were compared with the actual values obtained experimentally (Table 3). The absolute average difference was determined as the ratio of the average actual value to the predicted value, while the relative difference was determined as the absolute value of the ratio of the average actual and predicted values to the average actual value, according to the following formula:

(1) $$\delta =\frac{\left|x-x_{p}\right|}{\left|x\right|}\cdot 100\%$$

where *δ* is the average relative difference, *x* is the average actual (experimental) compressive strength, and *x_p_* is the predicted value based on the model.

Moreover, the RMSE (root mean square error) coefficient was analyzed, which represents the square root of the differences between the predicted values and the observed values and its value is normalized on the basis of the actual average value (coefficient of variation).

#### 3.2.2. The 0–6% Model

The model was based on the results of experimental research for uncontaminated and contaminated samples for the 0%, 1%, 2%, 4%, and 6% levels. Table 4 shows the obtained model parameters, along with the coefficient of determination R^2^, which describes the degree to which the models fit the actual data, while Table 5 shows the parameters of the exponential model obtained.

The compressive strength prediction accuracy of the models was considered in the 8% and 10% variant of contamination (Table 6).

### 3.3. Analysis of the Results

#### 3.3.1. Accuracy of Predictions Based on the Mathematical Model

**Figure 8:**
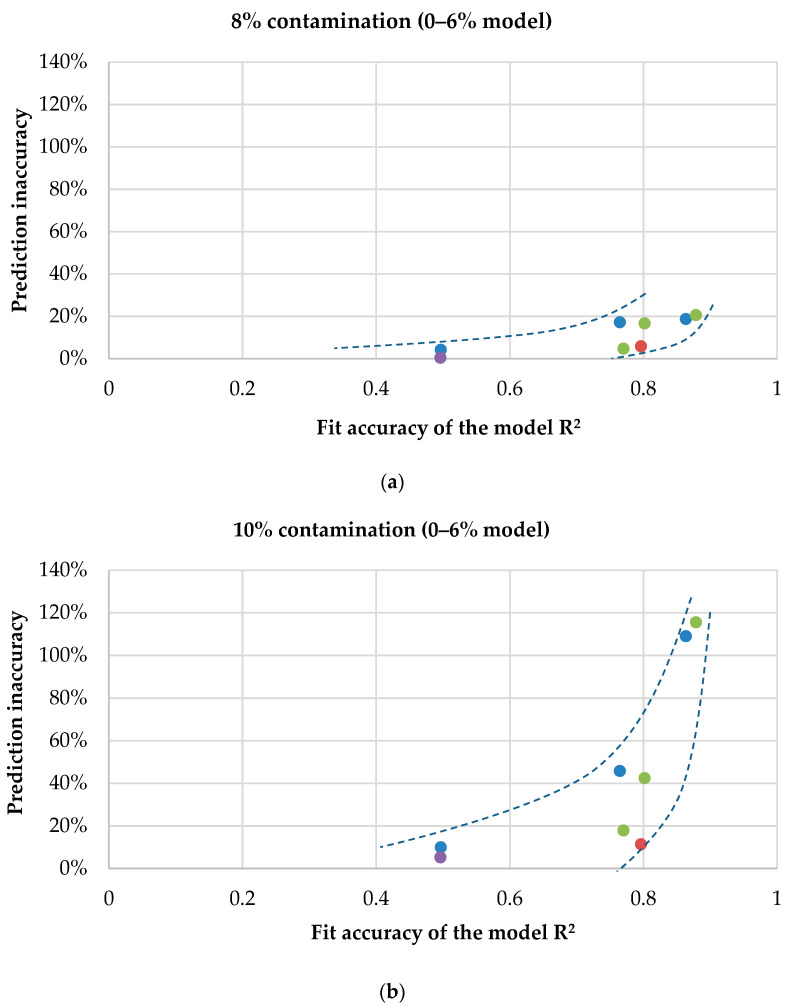

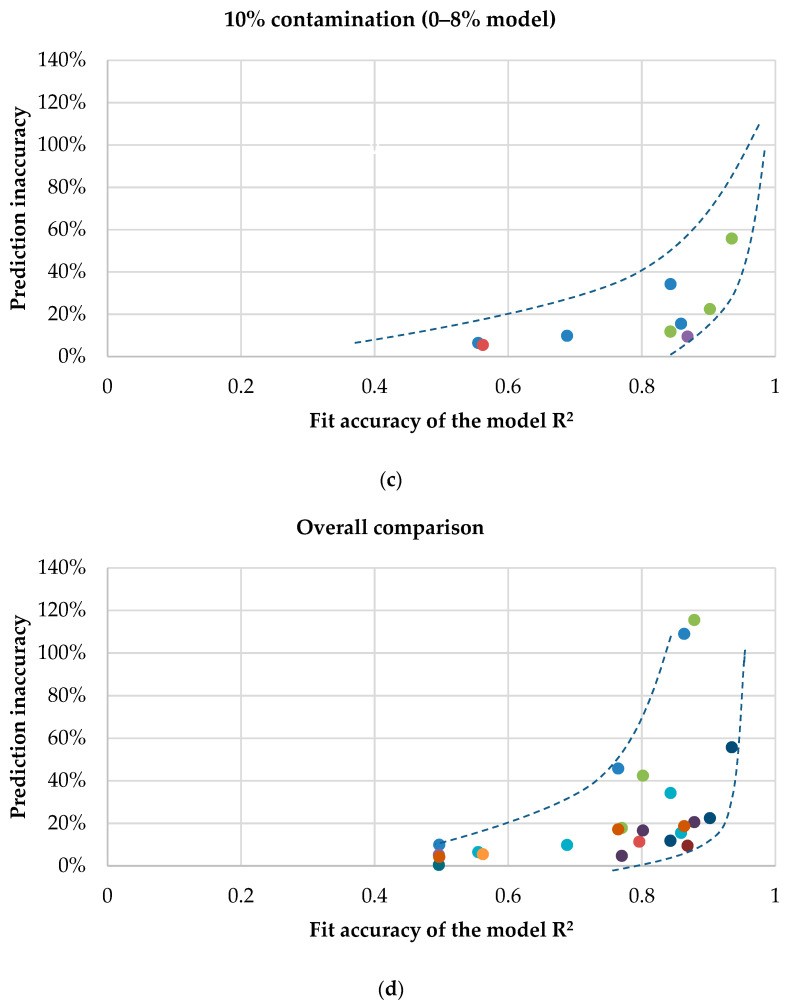
A comparison of R^2^ of the model and the absolute value of the degree of inaccuracy of the modeled compressive strength for different variants of contamination and different ranges of the teaching data for the model: (**a**) 8% contamination from the model taught in the range of 0–6%, (**b**) 10% contamination from the model taught in the range of 0–6%, (**c**) 10% contamination from the model taught in the range of 0–8%, and (**d**) an overall comparison.

A thorough analysis of the results obtained using statistical methods made it possible to notice some regularities resulting from the relationship between the accuracy of the fitting of the model to the entry data and the accuracy of the prediction of strength values determined based on this model in the event of an increase in the level of admixture of contaminants. Consequently, the model obtained from a smaller amount of data (0–6% range) allowed for quite precise determination of the strength values for blood or saline admixture level of 8% and 10%, but the difference between the values obtained in this way and the average experimental results for these variants was, depending on the selected model and the type of contaminant, from 0.16 MPa (99.8% of the average experimental value) to 75.45 MPa (213%). Some models also generated negative values for the compression strength of cements, which is obviously physically impossible. Interestingly, the coefficient of determination of the model that predicted a negative value reached R^2^ = 0.8634, and that of the abovementioned model, from which the prediction was made, was more than twice as high as the average actual value, R^2^ = 0.8785. The following conclusion was, therefore, drawn: In the case of mathematical modeling of the compressive strength of bone cements with admixtures of impurities, an increase in the accuracy of the fitting of the model is not accompanied by more accurate predictions of values outside the model range. Although the polynomial models of higher levels better fit the input data, their prediction of the values at 8% and 10% contamination levels turned out to be more inaccurate. Figure 8a–d shows an example of a comparison of R^2^ of the models and the percentage inaccuracy of estimation of the values.

Of note are the differences in the accuracy of determination (prediction) of values from different models. In the case of prediction of the compressive strength value for a sample with contamination higher by 2% than the extreme value of the contamination for which the model was generated, it is comparable in the variant of prediction of the strength of samples with 10% contamination from the model developed on the basis of samples contaminated in the range of 0–8% and samples with contamination of 8% from the model developed on the strength data for samples contaminated in the range of 0–6%. In both cases, the most accurate prediction was achieved in the linear and exponential models, which, in turn, were the worst with respect to fitting the data from which they were calculated.

On the other hand, much different in terms of accuracy were the strength prediction attempts for a significantly higher level of contamination, i.e., 10% from the model obtained from the data for the contamination range of 0–6%.

#### 3.3.2. Accuracy of Prediction on the Basis of an Artificial Neural Deep Learning Network

Analyses made with the use of an artificial neural deep learning network resulted in the determination of a time series and a sample destruction course, from which the predicted values of compression strength in the variant of 8% and 10% contamination of the cement structure with the two pollutants analyzed were read. The research was conducted in two ways: (1) by teaching and testing the networks with averaged courses of the actual process of compression of samples that were uncontaminated and contaminated with admixtures of 1–6% and 1–8% (for each level of contamination separately), which resulted in an averaged predicted course for the predicted variants, and (2) by generating several random combinations of samples (series), which were the materials that were used for teaching and testing the networks. Table 7 and Table 8 show a collective comparison of the obtained projected results and a reference to the actual results.

The selection of input data results in the change in prediction accuracy. An analysis of the abovementioned results clearly shows that out of the two methods used to teach the networks, results that were closer to the actual ones were obtained in the variant that involved creating random series from samples with different degrees of contamination. This observation requires confirmation in future research, perhaps on a wider or different range of input data. It is difficult to treat this individual observation as a basis for formulating recommendations as to which method of selecting data for this type of research is correct (or better) in general.

Unfortunately, only for the predictions of compression strength of cement with 8% contamination, the results from the networks, displayed an acceptable degree of inaccuracy (1.9% to 9.2%). Predicting the strength of cement with 10% contamination, regardless of the type of contamination, usually resulted in an inaccuracy of more than 12%. It is worth noting that the network always underestimated the results of the predicted strength compared to the actual samples. 

### 3.4. Comparison of the Results from Statistical Modeling and Deep Learning

**Figure 9:**
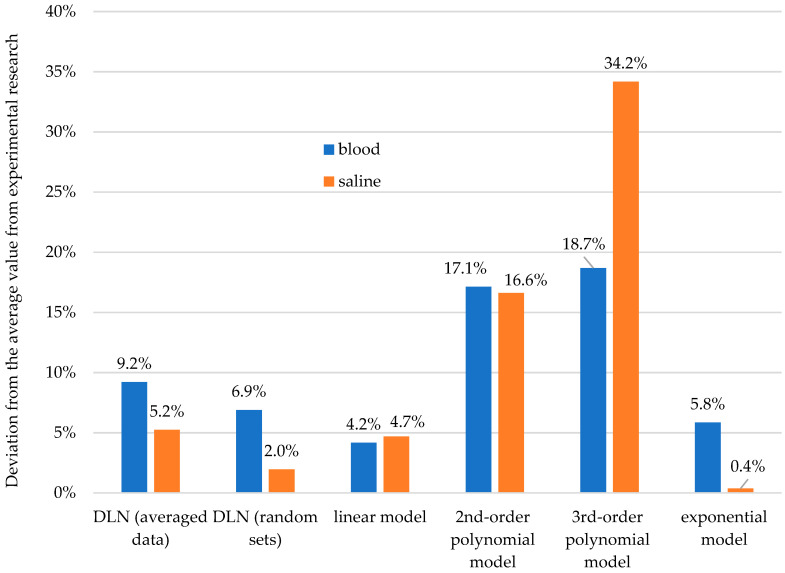
Uncertainty of prediction of strength at 8% contamination based on data from the 0–6% range.

Two fundamentally different methods of prediction for the behavior of bone cement material at different levels of contamination gave different numerical results. The most accurate prediction results were obtained by mathematical modeling of the function of strength changes in the variant that involved using experimental data from the 0–6% contamination range to predict the strength of cement with 8% contamination. They were obtained from the simplest linear model and the exponential model (Figure 9). Interestingly, of the examined models, it was the linear model that was most often characterized by the lowest value of fitting the teaching data (coefficient of determination). Analyses performed using artificial neural deep learning networks proved to be equally successful, regardless of how the teaching data were presented. The other polynomial models that were examined, despite a better degree of fitting (higher values of the coefficient of determination than for the linear model), predicted the effect of contamination on strength much less accurately. The results obtained at 15–35% deviation from the actual value, obtained experimentally, can hardly be considered to be useful.

**Figure 10:**
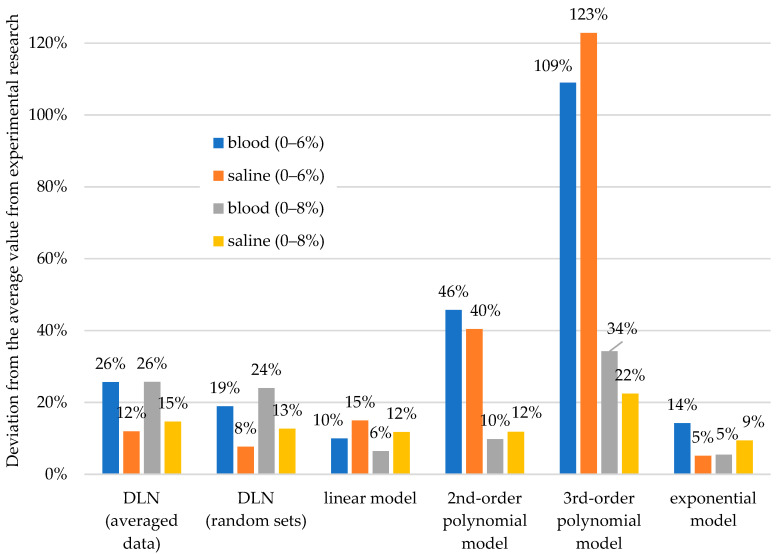
Uncertainty of strength predictions at 10% contamination based on the results obtained by different methods and from different ranges.

Slightly different analysis results were obtained in the case of prediction of the effect of 10% contamination. However, one should keep in mind that this attempt was made to predict a situation that was far beyond the range used for modeling and analysis using DLN (model data range: 0–6%, prediction: 10%). Interestingly, the results obtained from mathematical models developed from the strength data for contamination in the range of 0–6% (smaller amount) were not always less accurate than those generated from the data obtained at 0–8% contamination. Unfortunately, it is difficult to find any universal regularities here. In the variant of prediction from mathematical modeling, some models based on the 0–6% data were characterized by significantly lower accuracy than those based on the 0–8% data (3 to almost 6 times lower), which seems natural. Less data used to create the model resulted in lower usefulness for further prediction. In the case of models that gave better results in terms of fitting, e.g., the linear model, the deterioration was as follows: The fitting accuracy was 10% vs. 6% for blood and 15% vs. 12% for saline. Only in the case of the exponential model, for saline contamination, the model prepared on a smaller range of data better predicted the strength of cement with 10% contamination: 5% (the 0–6% model) vs. 9% (the 0–8% model) uncertainty. The analyses performed with the use of DLN were characterized by lower prediction accuracy in the case of blood and saline admixtures. The inaccuracies did not fall below 8–15% for saline and 19–26% for blood. A puzzling phenomenon is an inverse relationship between the larger range of data and the slightly higher values of deviation. The source of this phenomenon is the nature of DLN: Unlike classical artificial neural networks, deep learning networks are less attached to data. A summary of the inaccuracies of prediction of the values of compression strength of cements contaminated with 10% of saline by weight compared to the experimental results, based on various methods of analysis, is shown in Figure 10.

## 4. Discussion

Load transmission through the stem of a cemented THR joint depends on multiple factors, including the mechanical properties of the cement. An increase in compressive stress in the cement and on the cement/bone interface should lead to the long-term stability of the joint [13], but contamination of cement with blood or saline degrades its properties and may lead to joint damage with all the consequences this entails.

Methods for computer modeling methods of mechanical properties of biomaterials have great potential because they enable the preliminary estimation of the consequences of medical errors and often also a reduction of them. Knowledge of the scope of usefulness of the tested material may often protect patients from the need for repeated surgical interventions. This is also important from the point of view of physicians: Based on the estimated value of cement strength, they can present patients with probable scenarios of the outcome of the operation. This allows hospitals to reduce litigation costs in case of patient dissatisfaction.

The analysis of the reported results opens the way for further research related to the strength of biomaterials. The modeling results illustrate the fact and nature of a decrease in the strength of bone cements where contaminants are found. In the examined range of strength loss with an increase in the level of contamination, their close linear correlation was shown. Another important issue is the acceptable level of results from neural deep learning networks. This means that DLNs are suitable for predicting strength values and approximate characteristics, based on which the points determining the compressive strength of cement, which is the object of the research, were determined.

The analyses that were carried out show that a mathematical model that better fits actual data cannot always be used uncritically for precise predicting. In the case of bone cements, the prediction of a trend showing gradual changes in groups of strength characteristics proved to be quite well-described by means of a linear function. Due to the way neural networks work (predicting relationships in data difficult to describe with a single function and determining this function), there is a slight discrepancy between the results from random samples and the data from the combinations of these samples. Deep learning networks, compared to other types of networks in modeling mechanical properties (especially of materials with nonlinear strength characteristics), can be more effective due to the use of dropout (exclusion of certain neurons from the taught model during teaching, which reduces the interdependent fitting of neurons), which prevents the network from excessive fitting of the network to the training model. The advantages and disadvantages of using the network and statistical modeling are presented in Table 9.

Although relatively promising results were obtained, the process of predicting changes in the strength of bone cements using DLN networks is burdened with certain limitations. The application of DLN in the analyzed case may seem disproportionate to the size of the problem. The generalization capacity allows analyzing the issue in a much wider scope. It is expected that the capabilities of DLN could help study the phenomenon of bone cements’ strength in the case of post-operative contamination, due to corrosion at the tissue–cement interface and other concomitant factors. The applied tool could also be used in the analysis of the problems of cement fatigue strength correlating with the occurrence of material corrosion. The approach that involves modeling the entire strength characteristics with a DLN to find the point that constitutes (according to ISO 5833:2002 [35]) a destructive force, the load shifted by 2% or at the upper yield point (whichever occurred earlier), i.e., the strength characteristics of the material, may seem superfluous, but it paves the way for further works that will consider a substantially wider range of influencing factors. However, the nonstandard approach presented herein is aimed at extending the operation of strength analysis methods to include future research that will possibly also cover the plastic range and a full range of the strength characteristics as well as the research on the fatigue strength of contaminated bone cements. In conclusion, although the case of testing points that constitute the end of a linear range as a function of increasing admixture levels in the analyzed range can be easily described by means of a linear function, for a range extending beyond the analyzed admixture level area, the use of neural networks—including DLN—would be an effective solution, while a description with higher-level functions could work well for materials with nonlinear characteristics.

## 5. Conclusions

The generated higher-order polynomial models, despite their good fitting to the experimental data, are largely unsuitable for prediction purposes. Therefore, it is very important to wisely choose the type of model that we will use to try to describe the course of the changes. The model should result from observation of the nature of the variability of the data being modeled and should take into account the predicted boundary conditions. At the same time, it is important to make sure that the prediction range is reasonable. The further away our estimations are from the actual data, the greater the risk of errors and deviations from the actual values.

The results of contaminated bone cement compression strength predictions show that the use of DLNs for this purpose provides comparable results to those obtained with statistical modeling. However, very good models (linear, exponential) can give even more accurate results. It has been noted that the way networks are taught, i.e., the selection of input data, may slightly change the prediction accuracy. Due to the way neural networks work (predicting relationships based on data that is often difficult to describe with a single function, and determining this function), there is a slight discrepancy between the results obtained from random samples and the averaged data from the combinations of these samples. Thus, predicting based on the teaching of networks using a method that consists of a combination of random courses and then estimating the average value of the resultant courses usually gave more accurate results (the deviation from the average, experimental value: 2% saline, 7% blood) in comparison to the usage of averaged input data (5% saline, 9% blood), as presented in Figure 9. The difference was about 2–3%. The biggest disadvantage of employing DLN in the presented research is the use of its relatively high computing power for an indirect prediction of the compressive strength (one point in the time course).

From the results reported here, it can be concluded that the general direction of further research should extend the scope of analyses presented in this paper. It would be interesting to confirm the observed change in prediction accuracy depending on how the teaching set for DLN is organized. Also, the authors plan to test the presented methods on data collected in similar research on different mechanical properties of biomaterials and compare their predictive strength.

Based on the presented results, it is possible to set out to construct an expert system. After accepting a given network, generating its structure with a complete processing algorithm, a system would be built to support the assessment, here, of the impact of a potential degree of contamination on the expected cement strength. After supplementing the existing database with new data, e.g., a higher degree of contamination, a system based on the generated information-processing algorithm would assess the potential strength of the cement with a new set of data.

## Figures and Tables

**Table 1:** Parameters of selected polynomial models—α_1_x^4^ + α_2_x^3^ + α_3_x^2^ + a_4_x + ε—obtained from 0–8% data.

Model Type	α_1_	α_2_	α_3_	α_4_	ε	R^2^
blood 1°	-	-	-	−140.97	79.843	0.5552
blood 2°	-	-	3,047.6	−388.31	82.203	0.6881
blood 3°	-	−147,053	21,125	−925.41	84.218	0.8431
blood 4°	2,662,604	−572,121	41,532.17	−1,210.6	84.524	0.8588
saline 1°	-	-	-	−272.31	88.626	0.8428
saline 2°	-	-	-23.8	−270.27	88.606	0.8428
saline 3°	-	129,323	-16,919	266.04	86.282	0.9019
saline 4°	−4,944,022	965,912	-59,549.84	906.92	85.216	0.9348

**Table 2:** Parameters of exponential models—y = K_0_e^kx^—obtained from the 0–8% data.

Model Type	K_0_	k	R^2^
blood exp.	79.705	−1.85	0.562
saline exp.	88.924	−3.537	0.8684

**Table 3:** Prediction accuracy of selected models for the case with 10% admixture from the 0–8% model.

Model Type	Predicted Value 10%	Average Actual Value 10%	Average Absolute Difference	Average Relative Difference	RMSE	CV (RMSE)
blood 1°	64.62	69.05	4.43	6%	5.14	8%
blood 2°	75.81		6.76	10%	7.24	10%
blood 3°	45.43		23.62	34%	23.76	52%
blood 4°	79.75		10.70	15%	11.01	14%
blood exp.	65.65		3.78	5%	4.59	7%
saline 1°	58.91	66.77	7.82	12%	7.87	13%
saline 2°	59.40		7.90	12%	7.95	13%
saline 3°	80.47		14.98	22%	15.00	18%
saline 4°	29.55		37.21	56%	37.22	126%
saline exp.	60.44		6.29	9%	6.34	10%

**Table 4:** Parameters of selected polynomial models—α_1_x^3^ + α_2_x^2^ + α_3_x + ε—obtained from the 0–6% data.

Model Type	α_1_	α_2_	α_3_	ε	R^2^
blood 1°	-	-	−167.86	80.32	0.4968
blood 2°	-	7093.8	−606.42	83.38	0.7647
blood 3°	−264,373	31,084	−1113.4	84.528	0.8634
saline 1°	-	-	−299.3	89.107	0.7701
saline 2°	-	−3,350.5	−79.742	87.471	0.8016
saline 3°	299,365	−32,258	577.4	85.571	0.8785

**Table 5:** Parameters of exponential models—y = K_0_e^kx^—obtained from the 0–6% data.

Model Type	K_0_	k	R^2^
blood exp.	89.354	−3.808	0.7963
saline exp.	80.144	−2.16	0.4961

**Table 6:** Prediction accuracy of selected models for (**a**) 8% and (**b**) 10% admixture from the 0–8% model.

(**a**)						
**Model Type**	**Predicted Value 8%**	**Average Actual Value 8%**	**Average Absolute Difference**	**Average Relative Difference**	**RMSE**	**CV (RMSE)**
blood **1°**	66.56	69.45	−2.89	4%	2.72	4%
blood 2°	81.35		11.90	17%	12.83	16%
blood 3°	56.47		−12.98	19%	12.30	22%
blood exp.	65.39		4.06	6%	3.69	6%
saline 1°	63.07	66.17	−3.10	5%	3.14	5%
saline 2°	55.17		−11.00	17%	11.01	20%
saline 3°	88.78		22.61	21%	22.61	25%
saline exp.	66.41		0.25	0%	0.54	1%
(**b**)						
**Model Type**	**Predicted Value 10%**	**Average Actual Value 10%**	**Average Absolute Difference**	**Average Relative Difference**	**RMSE**	**CV (RMSE)**
blood 1°	62.19	69.05	6.86	10%	7.33	12%
blood 2°	100.63		31.58	46%	31.68	31%
blood 3°	−6.19 *		75.24 *	109% *	75.28 *	−1.216% *
blood exp.	59.22		9.83	11%	10.16	17%
saline 1°	56.78	66.77	9.99	8%	10.02	18%
saline 2°	39.78		26.99	42%	27.00	68%
saline 3°	148.79		82.02	115%	82.02	55%
saline exp.	63.33		3.44	5%	3.53	6%

* Negative value of predicted compression strength.

**Table 7:** Compressive strength prediction accuracy for DLN taught in the range of 0–6%: (**a**) 8% contamination and (**b**) 10% contamination.

(**a**)				
**Contamination Dataset**	**Predicted Value 8%**	**Average Actual Value 8%**	**Absolute Difference**	**Relative Difference**
blood—averaged	63.05	69.45	6.40	9.2%
blood—random series	64.66		4.79	6.9%
saline—averaged	62.70	66.17	3.47	5.2%
saline—random series	64.88		1.29	1.9%
(**b**)				
**Contamination Dataset**	**Predicted Value 10%**	**Average Actual Value 10%**	**Absolute Difference**	**Relative Difference**
blood—averaged	51.34	69.05	17.71	25.6%
blood—random series	55.98		13.07	18.9%
saline—averaged	58.78	66.77	7.99	12.0%
saline—random series	61.65		5.12	7.7%

**Table 8:** Compressive strength prediction accuracy for DLN taught in the range of 0–8% of contamination.

Contamination Dataset	Predicted Value 10%	Average Actual Value 10%	Absolute Difference	Relative Difference
blood—averaged	51.30	69.05	17.75	25.7%
blood—random series	52.49		16.56	24.0%
saline—averaged	56.98	66.77	9.79	14.7%
saline—random series	58.33		8.44	12.6%

**Table 9:** Comparison of deep learning networks and statistical analysis advantages and drawbacks.

DLN	Statistical Analysis
Requires special tools	Can be conducted using dedicated software or simple spreadsheets (Microsoft Excel or similar)
Often requires expensive software	Freeware/open software can be used
Requires knowledge in the area of general DLN theory and the ability to work in a specific program	Requires medium statistical knowledge and basic computer skills
The results are satisfactory	The results depend heavily on the selected model and its fit to data
Possibility of generalization for unknown cases	Limited possibilities of problem generalization (possibility of reliable forecasting in the analyzed range)
